# Antecedents and Consequences of Therapeutic Communication in Iranian Nursing Students: A Qualitative Research

**DOI:** 10.1155/2017/4823723

**Published:** 2017-12-13

**Authors:** Mahbobeh Abdolrahimi, Shahrzad Ghiyasvandian, Masoumeh Zakerimoghadam, Abbas Ebadi

**Affiliations:** ^1^Medical-Surgical Nursing Department, School of Nursing and Midwifery, Tehran University of Medical Sciences, Tehran, Iran; ^2^Critical Care Nursing Department, School of Nursing and Midwifery, Tehran University of Medical Sciences, Tehran, Iran; ^3^Behavioral Sciences Research Center (BSRC), Nursing Faculty, Baqiyatallah University of Medical Sciences, Tehran, Iran

## Abstract

In recent years, particular attention has been paid to nursing students' therapeutic communication (TC) with patients, due to a strong emphasis on patient-centered education in the Iranian healthcare reform. However, various studies have highlighted the poor communication of future nurses. Therefore, researchers have used qualitative methodology to shed light on the antecedents and consequences of nursing students' TC and promote it. We carried out a conventional content analysis using semistructured interviews with a purposefully selected sample of 18 participants, including nursing instructors, students, and patients in hospitals affiliated to Tehran University of Medical Sciences. “Communication readiness,” “predisposing factors,” and “continuity of care” were identified as the three major themes. “Communication readiness” consisted of “physical readiness,” “academic readiness,” and “developmental readiness.” “Predisposing factors” included “contextual factors” and “educational condition.” “Continuity of care” included “patient satisfaction” and “improving nursing student's motivation to communicate with patients.” “Communication readiness” and “predisposing factors” constitute the antecedents of nursing student's TC with patients, and “continuity of care” is considered as its consequence. More attention needs to be paid by the regulators to TC instruction in both theoretical and clinical educational curriculum. Furthermore, all nurses must be informed about the importance of TC in promoting patient outcomes and quality of care.

## 1. Introduction

Communication, considered as an important subject by many multidisciplinary scholars, is defined as the transfer of messages containing information and feeling among participants to satisfy basic social human needs [1]. Healthcare providers need to communicate with their clients, based on Hippocrates' medical principles, more than any other professionals [2]. Nurses, who are the largest group of healthcare providers, have to communicate with coworkers, physicians, paramedics, and patients in order to effectively fulfill their complex roles [3]. However, due to lengthy encounters between nurses and clients [4], and also to comply with Iranian national healthcare reform requiring patient education for providing healthcare for all Iranians by 2025 [5], therapeutic communication (TC) between nurses and patients is considered as one of the most significant clinical communications [6]. TC is defined as the process of applying verbal or nonverbal communication to connect with patients in order to recognize their problems and help them to understand how they should take care of themselves [7]. Nursing students as one of the first healthcare providers who work closely with patients must learn to be effective communicators [8]. Therefore, the concept of TC has been incorporated into the nursing curriculum to meet the students' educational needs as well as patients' healthcare demands [9]. In Iran, effective communication is taught through theoretical lectures by nursing instructors and is evaluated through written exams [10]. However, some studies have indicated that education cannot guarantee the development of TC in nursing students, and they could not communicate effectively with patients [3, 11, 12]. This demonstrates the necessity of considering this concept more seriously during formal academic training [10]. TC, which is strongly affected by different stakeholders including nursing students, patients, and nursing instructors, is a complicated and context-based concept and needs to be disambiguated for better clinical instruction [1, 7, 12].

## 2. Material and Methods

The present study aimed to provide an insight into what makes Iranian nursing students effective in TC and what benefits it has for nursing students as well as patients in the hospitals affiliated with Tehran University of Medical Sciences in 2016-2017. In this study, an exploratory qualitative approach was chosen. The main research questions were as follows: “What factors help nursing students to build TC with the patient?” and “What possible outcomes would result when a nursing student and a patient establish TC?”

The first author, a Ph.D. candidate in nursing with adequate clinical teaching experience and who had participated in various workshops about qualitative data gathering and analysis, held semistructured, face-to-face interviews with the participants to collect data [13]. The researcher chose participants who had rich experiences of TC and selected them through a purposeful sampling after spending some time in the study environment taking into account the principles of maximum variation [14]. After a warm-up, willing participants who could speak Farsi fluently were interviewed at a convenient time and in a quiet place. Six second, third, and fourth year BSc nursing students (who had passed at least one clinical rotation in hospitals affiliated to the University of Medical Sciences), six alert patients (being cared for by nursing students for at least five days), and six nursing educators (affiliated with the School of Nursing and Midwifery) participated in this study. An interview guide which included open-ended questions designed after preliminary interviews with four nursing students and patients was used by the interviewer. Interviews, which were audio-taped with a voice recorder, continued based on the participants' responses, and probing questions were used to obtain a clear response [13]. The interviewer wrote field notes in order to consider the different emotions and behaviors of the participants. The mean length of interviews was 40 min.

Using MAXQDA-12 software, the researchers adopted inductive conventional content analysis, which is one of the most common methods used in qualitative studies for data analysis. According to Zhang and Wildemuth, this method of data interpretation includes seven steps. In the first step (data preparation), the audio-taped interviews were typed verbatim. In the second, four research team members studied the texts several times to determine the semantic units. In the third, the units were abstracted and labeled as codes, and similar codes were classified into subcategories. Then, the similar subcategories were combined to form categories. In the fourth, the codes were tested by the researchers on a part of the text. In the fifth, the researchers coded the entire interview. In the sixth, after ensuring the data consistency, the researchers applied thematic analysis to generate themes from categories. Finally, in the seventh step, the data were reported [15].

Guba and Lincoln's criteria were adopted to evaluate the quality of data. To ensure data credibility from the participant's point of view, the researchers returned the transcripts and codes to the participants and modified the original data on the basis of their comments. Furthermore, the researchers used maximum variation sampling (participants of different ages, genders, and working experience) to obtain a heterogeneous sample of participants. A panel of six experts in the field of communication commented on the research rigor. To ensure data transferability, the researchers conducted a thorough data documentation regarding the details of the investigation that would help other scholars to understand and conduct similar studies in other contexts. In addition to the reporting of methods of data collection and interpretation, the researchers used prolonged sampling (12 months) and simultaneous data interpretation to maintain data dependability. The researchers attempted to establish data confirmability by gathering different participants' points of view as well as reaching a consensus among interpreters [14].

The researcher obtained permission from the vice-chancellor and Ethics Committee of the University of Medical Science and managers of affiliated hospitals to enter the research field. Before data collection, the researchers informed all interviewees about the research aims, their voluntary participation, data confidentiality, and audio-taping of their voices, in compliance with the ethical principles of Tehran University of Medical Sciences. Then, a written informed consent form was signed by the participants.

## 3. Results

After the 18th interview, no new codes were extracted from the interviews, and data saturation was reached. Half of the participants were males. The average age of the nursing students, instructors, and patients was 21.50, 36.83, and 56.50 years, respectively. The instructors' mean university teaching experience was 15.5 years. The average length of patients' hospital stay was 7 days. The participants' demographic characteristics are shown in Table 1.

In total, 34 codes, 14 subcategories, and 7 categories were obtained from the data analysis. After thematic analysis, three themes emerged, including “communication readiness,” “predisposing factors,” and “continuity of care.” The major themes of TC and its corresponding categories are presented in Table 2.

### 3.1. Communication Readiness

The first major theme, “communication readiness,” was classified into three categories of “physical readiness,” “academic readiness,” and “developmental readiness.” The first category, “physical readiness,” consisted of two subcategories: “patient's patience” and “nursing students' power to communicate with patients.” A student (7) stated the following regarding a patient's impatience:* “a patient was very angry due to having continued urinary tract problems for 2 weeks after hospitalization. Therefore, we could not take her history.” *

Moreover, nursing students must have enough energy to interact with patients therapeutically. Another student (11) highlighted the following:* “I can't get a dorm room in campus. Moreover, I don't have enough energy to interact with patients after a night shift.”*

Nursing students' “academic readiness,” which includes adequate “knowledge and skills in nursing,” influences their interactions. In this regard, a participant (12) said,* “when we have broad knowledge of nursing as well as clinical communication experience, we can provide patients with more information.” *TC instruction and assessment by nursing instructors are important factors in trainees' readiness to establish TC. A participant (17) stated,* “I teach communication skills to freshman students practically. Moreover, I include TC in the final exam.”*

The category of “developmental readiness” was summarized in two subcategories of “personality characteristics” and “motivation.” With regard to the role of nursing students' personality characteristics in TC, an instructor (14) stated the following:* “in addition to the necessity of having a high self-esteem, nursing students must be extroverted to console patients by demonstrating empathy.” *Furthermore, a trainee (9) stated that nursing students' clinical position and their attitude toward nursing affect their motivation to create effective interaction:* “If trainees believe in nursing as a valuable job, they will interact with the patient effectively. Students don't have a place in which to sit in the wards, and this decreases their motivation for TC.”*

### 3.2. Predisposing Factors

This theme included two categories: “contextual factors” and “educational conditions.” The category of “contextual factors” contained two subcategories, including “cultural values” and “professional rules.” One of the patients (4) stated the following regarding cultural values:* “English patients ask personnel many questions before every procedure. But we Iranians totally trust the staff and accept everything they say to us without pondering about it.” *Furthermore, a patient (3) about patients' preference for receiving care from a nursing student of the same gender stated that* “if I have a health professional with the same gender looking after me, I will trust them more easily.” *Another patient (6) told the interviewer about the effect of family education on TC:* “some of the students haven't learned how to speak politely with a person in their families.” *Also, about the necessity of establishing TC as a religious and professional duty, a participant (15) stated that* “saying hello, the basis of effective communication, is recommended in Islamic rules.” *Moreover, another instructor (18) said,* “TC is a professional responsibility that we nurses must perform.”*

The “educational condition” category consisted of two subcategories: “the necessity for a suitable educational hospital” and “appropriate educational regulations.” An instructor (13), regarding the support of staffs in suitable educational hospital, stated the following:* “support of staffs helps us to communicate comfortably with patients in a secure place.” *Also, nursing teachers must have a positive attitude toward TC to instruct trainees properly. A participant (1) stated,* “some teachers don't have a positive attitude toward TC to motivate us to interact with our patients.” *Another participant (16), regarding the nursing instructors' knowledge deficit, stated that* “some nursing teachers don't have the required knowledge to answer our questions. Therefore, the personnel and patients don't trust them.” *A participant (5), about the necessity of empowering nurses in TC, stated that* “The nurse's knowledge of communication wasn't enough and he couldn't manage the ill-tempered patient.”* Appropriate educational regulations should consider TC for training a nurse who can work based on the international standards of care. A participant (7) told the researcher:* “they assign one instructor for 10 nursing students at a 5-day clinical placement. They should decrease the number of students and increase the length of the clinical placement.” *Also, a student (8) talked about the significance of determining nursing students' uniform dress:* “I think nursing students should have a special uniform so that patients do not consider them as a nutritionist or a doctor.”*

### 3.3. Continuity of Care

The last major theme was “continuity of care,” which contained two categories: “patient satisfaction” and “improving nursing students' motivation to communicate with patients.” “Patient satisfaction” was classified into two subcategories of “patient comfort” and “patients' physical health.” A patient (2) told the researcher about his composure after the interaction:* “when the trainee established emphatic interaction with me, I trusted him and my stress levels decreased.” *Another participant (10) stated the following with regard to the benefits of TC for patients' physical health:* “when I conduct TC, patients tell me about their private physical problems. Then, I quickly develop a practical care plan to solve their problems.” *Moreover, patients' readmission decreases with patient education. A patient (6) recounted the following:* “had they told me what to do in my previous hospitalization, I wouldn't have been here once again.”*

The “improving nursing students' motivation to communicate with patients” category was divided into two subcategories of “completing nursing students' assignments” and “comfortable provision of healthcare.” Nursing students communicate with their patients to meet their educational requirements. A student (11) said,* “students ask patients so many questions for patient assessment and history taking.” *Moreover, the use of TC facilitates healthcare provision. A participant (9) told the researcher the following:* “after establishing effective communication, I deliver professional healthcare more calmly and comfortably.”*

## 4. Discussion

Hospital accreditation emphasizes the importance of having qualified nurses to perform TC [16]. To promote the effectiveness of nursing students' encounters with patients, the researchers decided to investigate the antecedents and consequences of Iranian nursing students' TC through exploration of various stakeholders' perceptions.

Regarding the physical readiness, some factors such as the severity of patients' illness and long hospitalizations have a negative impact on patients' moods as they interrupt communication. This finding is similar to the results of a study about the change of mood and communication along with physical problems during patient hospitalization in the United States [17]. Also, nurses' physical and psychological fatigue after working for long hours had negative effect on their performance and decreased patient satisfaction [18].

Regarding the academic readiness, theoretical knowledge and clinical communication skills helped nursing students to interact effectively with their patients. These skills are the most important facilitators that help to gain patients' trust and develop trainees' self-confidence, which leads to nursing students' success in the clinical environment [19]. Considering the TC as an imaginative art rather than a skill or a subjective concept [20], some researchers suggest that adopting creative, learner-centered, and practical approaches such as cooperative and competency-based methods enhance trainees' meaningful learning of TC skills [7, 9, 10]. However, Iranian nursing instructors, like some other Asian educators, use the traditional teacher-centered models to teach TC that continues causing problems in healthcare provision [10, 21].

Regarding the developmental readiness, personality traits such as an extraversion personality and having a high self-esteem, along with trainees' positive belief about TC which is influenced by their interest in nursing, as well as students' proper position in the clinical settings, affect the acceptance of patients with various sociocultural characteristics [12, 22].

Regarding contextual factors, being of the same gender as that of the patient is a key factor in building trust between clients and caregiver. However, this could be due to the Iranian Islamic culture, but it is consistent with an American research which found that the male gender of paramedical technicians resulted in patients refusing prehospital care [23]. Furthermore, Iranian patients would trust nursing student after perceiving that trainees intend to support them through effective and correct patient education which is in line with another Iranian research [24]. Moreover, the lack of emphasis during the upbringing of students on the subject of respecting the dignity of older people has resulted in the creation of a new generation of nursing students who do not regard ethical principles of interaction. Family education and preparing social people through teachings by elder family members are widely recognized as an important issue in modern Chinese literature [25]. Also, another Iranian content analysis study demonstrated that there is moral distress in intensive care units, which can lead to personnel's burnout [26].

In the present research, some interviewees referred to TC as a professional and religious duty. One of the most important duties of a professional healthcare provider is to consider both verbal and nonverbal messages of patients to understand their messages completely [6]. Furthermore, in another study, some Iranian nurses who strongly believed in spiritual care and Allah communicated successfully and more effectively with patients' families to give them hope and encouraged them to pray for their loved ones [27].

A good educational hospital, with capable nurses and nursing instructors as role models, provided trainees with an appropriate environment to help them achieve better educational results and develop communication skills, which is consistent with the result of a review study regarding international medical students' learning in the clinical setting [28]. Thus, hospitals' educational departments, as well as nursing schools, must empower nurses and nursing instructors in TC to prepare them for their communicative roles [19]. Furthermore, nursing instructors with adequate knowledge and positive attitudes toward TC can teach and motivate trainees to uphold TC with patients by using measures such as considering students' interaction in the final exam to improve students' practical learning, which is in agreement with other Iranian studies [12, 29]. Moreover, enabling nurses in TC through continuous education is recommended as it improves the quality of nurses' interactions with patients in multicultural countries such as Iran [3, 30] and the United States [31].

Educational rules and regulations should consider incorporating theoretical and practical courses in TC in the B.S. nursing curriculum [2], reduce the number of students in the more extended clinical rotations [21], and determine the professional dress code for nursing students [7].

The results also showed that study participants talked about TC antecedents more than consequences as they had less knowledge about the outcomes of effective communication, which decreased their motivation to establish TC. Effective interaction leads to resolution of the patient's problem [10], reduction of patient's stress [3, 12, 21, 30], and creation of an empathic relationship between the student and the patient [3, 9, 12, 21], which increase patient's satisfaction [12] and cooperation in the caring process [9]. Moreover, in this study, nursing students used TC to develop an evidence-based treatment plan and provide patients with education to meet their educational requirements [3, 8], which can lead to patient autonomy [7] and fewer hospital admissions [30]. TC helps to raise nursing students' motivation to connect with patients besides making healthcare provision easier and more comfortable. This result is in agreement with that of other studies in which communication skill education facilitated students' professional socialization [12] and boosted their confidence to deliver safe and quality care [3, 7, 21].

Although researchers examined various experiences of the participants regarding TC in the hospitals affiliated to the largest medical university in the country, less is known about the nursing student-patient TC experiences in other university hospitals. Therefore, further studies are required to investigate this concept in other provinces of the country.

## 5. Conclusion

By studying the data, the researchers considered “communication readiness” and “contextual factors” as nursing students' TC antecedents and “continuity of care” as its consequence. By using TC antecedents, nursing instructors may be more successful in facilitating trainees' interactions. Furthermore, both clinical managers and instructors can use the consequences of the concept to encourage nurses and nursing trainees to establish TC in clinical settings to achieve desired outcomes for both the patients and the healthcare providers. Educational managers can also apply these results to make amendments to the theoretical and clinical nursing education curriculum and give more weight to student-centered teaching methods, including simulation and self-evaluation, for more training of nurses with basic communication skills.

## Figures and Tables

**Table 1:** Demographic characteristics of the participants.

Code	Participant	Education level	Gender	Age (year)	Teaching experience (year)	Duration of hospitalization (day)
(1)	Patient 1	Bachelor's degree	Female	60		13
(2)	Patient 2	High school diploma	Male	35		8
(3)	Patient 3	Primary school	Male	68		8
(4)	Patient 4	Primary school	Female	49		5
(5)	Patient 5	B.S. student	Female	30		2
(6)	Patient 6	High school diploma	Male	50		6
(7)	Student 1	Third semester	Female	21		
(8)	Student 2	Fourth semester	Female	20		
(9)	Student 3	Fifth semester	Male	22		
(10)	Student 4	Sixth semester	Female	21		
(11)	Student 5	Seventh semester	Male	22		
(12)	Student 6	Eighth semester	Male	23		
(13)	Instructor 1	Master's degree	Female	47	23	
(14)	Instructor 2	Master's degree	Male	56	25	
(15)	Instructor 3	Ph.D.	Female	37	10	
(16)	Instructor 4	Bachelor's degree	Male	49	25	
(17)	Instructor 5	M.S. student	Male	32	6	
(18)	Instructor 6	Ph.D. student	Female	31	4	

**Table 2:** The major themes of therapeutic communication and its corresponding categories.

Subcategory	Category	Theme
Patient's patience	Physical readiness	Communication readiness
Nursing students' power to communicate with patients		
Personality characteristics	Developmental readiness	
Motivation		
Having adequate knowledge	Academic readiness	
Having sufficient skill in performing therapeutic communication		
Influences of cultural values on communication	Contextual factors	Predisposing factors
Impact of professional rules on communication		
The necessity of a suitable educational hospital	Educational condition	
Appropriate educational regulations		
Patient comfort	Patient satisfaction	Continuity of care
Improving patient's physical health		
Completing nursing students' assignments	Improving nursing students' motivation to communicate with patients	
Comfortable provision of healthcare due to improved position of nursing students in the society and their increased self-esteem		
